# Commentary: Torpor: The Rise and Fall of 3-Monoiodothyronamine from Brain to Gut—From Gut to Brain?

**DOI:** 10.3389/fendo.2017.00206

**Published:** 2017-08-22

**Authors:** Annunziatina Laurino, Laura Raimondi

**Affiliations:** ^1^Department of Psychology, Neurology, Drug Sciences, Health of the Child, Pharmacology, Section of Pharmacology and Toxicology, University of Florence, Florence, Italy

**Keywords:** 3-iodothyronamine, 3-iodothyroacetic acid, histamine, hypothermia, trace amines, thyroid hormone derivatives

In this issue of Frontiers in Endocrinolgy, Glossman and Lutz present an interesting review of the pharmacological effects of 3-iodothyronamine (T1AM) reported in literature questioning the relevance of T1AM hibernating activity. For the first time, pharmacological effects of the amine are discussed in the context of its pharmacodynamic and pharmacokinetic properties. The authors’ analysis reveals discrepancies and possible misinterpretation of results which might have led to an overestimation of T1AM pharmacological profile.

Glossman and Lutz smartly discuss, in particular, the reasons why T1AM cannot be proposed as an hibernating agent pointing the reader’s attention to the following simple observations: (i) the torpor like effect is obtained administering very high doses of T1AM, (ii) it is known that T1AM, is a multi-target molecule, in virtue of its chemical structure, and the hibernating effect cannot result from activation of trace amine associated receptors, and (iii) because of its poor pharmacokinetic properties, T1AM needs to be administered at high doses, inevitably exposing the subject to the risk of side effects. Thus, Glossman and Lutz conclude that T1AM would not merit to be further explored as a torpor-inducing drug or considered as a led compound for the synthesis of novel hibernating drugs.

Since our laboratory has been studying the pharmacological properties of T1AM and its oxidative metabolite, the 3-iodothyroacetic acid (TA1), the paper by Glossman and Lutz was very interesting to us and we felt compelled to add a new spin to their review.

Before discarding any knowledge surrounding T1AM, we would like to underline some recent studies that suggest that pharmacokinetic is essential for the manifestation of T1AM pharmacological effects when administered at very low doses (μg/kg). The evidence provided indicates that the observed T1AM properties as a potent learning, memory and curiosity enhancer (1), and a modifier of mice feeding are dependent on its oxidative deamination (2). Consistently, administration of TA1, the main T1AM metabolite (3), reproduced in mice most of the same effects described for T1AM, with similar inverted U-shaped dose-effect curves. Even though, the target(s) responsible for T1AM, or TA1, effects still remain elusive, we found evidence for the involvement of the histaminergic system in T1AM and TA1-induced behavioral effects. Although, the activation histaminergic system may account for unpleasant effects, including itchiness, experimental evidence indicates that this effect does not occur when a higher dose of TA1 is used, i.e., the same dose required for the compound to be active on memory (4, 5). In short, TA1 selectively recruits different mechanisms depending on the dose administered, such that effects observed at doses as low as 0.4 µg/kg are not evident when a higher dose of 1.32 µg/kg is used. The pharmacological meaning of such differences is still unknown and likely involves the nature of the molecule and of the target(s) activated including desensitization mechanisms.

Overall, this evidence indicates that T1AM metabolism does not abolish its pharmacological effects, as would be expected for a trace amine, but rather it could be responsible for the local availability of TA1 (since mono amine oxidases are ubiquitous intracellular non-microsomal phase-I enzymes), which is thought to be the active principle of T1AM.

If this hypothesis could be conclusively demonstrated by basic research, a new scenario would open where TA1 is the drug, while T1AM is the prodrug, whose pharmacokinetic is critical for making TA1 available locally.

## Author Contributions

AL and LR discussed the topic and wrote the paper.

## Conflict of Interest Statement

The authors declare that the research was conducted in the absence of any commercial or financial relationships that could be construed as a potential conflict of interest.
